# Projection of future pharmacy service fees using the dispensing claims in hospital and clinic outpatient pharmacies: national health insurance database between 2006 and 2012

**DOI:** 10.1186/s12913-018-3067-0

**Published:** 2018-05-03

**Authors:** Dongmun Ha, Inmyung Song, Eui-Kyung Lee, Ju-Young Shin

**Affiliations:** School of Pharmacy, Sungkyunkwan University, 2066 Seobu-ro, Jangan-gu, Suwon, 16419 South Korea

**Keywords:** Pharmacy service fee, Drugs cost, Hospital outpatient pharmacy, Clinical outpatient pharmacy, Prediction model

## Abstract

**Background:**

Predicting pharmacy service fees is crucial to sustain the health insurance budget and maintain pharmacy management. However, there is no evidence on how to predict pharmacy service fees at the population level. This study compares the status of pharmacy services and constructs regression model to project annual pharmacy service fees in Korea.

**Methods:**

We conducted a time-series analysis by using sample data from the national health insurance database from 2006 and 2012. To reflect the latest trend, we categorized pharmacies into general hospital, special hospital, and clinic outpatient pharmacies based on the major source of service fees, using a 1% sample of the 2012 data. We estimated the daily number of prescriptions, pharmacy service fees, and drugs costs according to these three types of pharmacy services. To forecast pharmacy service fees, a regression model was constructed to estimate annual fees in the following year (2013). The dependent variable was pharmacy service fees and the independent variables were the number of prescriptions and service fees per pharmacy, ratio of patients (≥ 65 years), conversion factor, change of policy, and types of pharmacy services.

**Results:**

Among the 21,283 pharmacies identified, 5.0% (1064), 4.6% (974), and 77.5% (16,340) were general hospital, special hospital, and clinic outpatient pharmacies, respectively, in 2012. General hospital pharmacies showed a higher daily number of prescriptions (111.9), higher pharmacy service fees ($25,546,342), and higher annual drugs costs ($215,728,000) per pharmacy than any other pharmacy (*p* <  0.05). The regression model to project found the ratio of patients aged 65 years and older and the conversion factor to be associated with an increase in pharmacy service fees. It also estimated the future rate of increase in pharmacy service fees to be between 3.1% and 7.8%.

**Conclusions:**

General hospital outpatient pharmacies spent more on annual pharmacy service fees than any other type of pharmacy. The forecast of annual pharmacy service fees in Korea was similar to that of Australia, but not that of the United Kingdom.

## Background

The accurate prediction of future medical expenditure can inform policy debates and decision making regarding healthcare resource allocation [1, 2]. Likewise, predicting pharmacy spending, a major factor of national health financing, is essential for budget planning [3]. Forecasting pharmacy service fees, in particular, can be important to sustain the health insurance budget from the payer’s perspective while maintaining pharmacy management. Several studies have used statistical modelling approaches based on pharmacy insurance claims data to predict annual healthcare costs in the United States and Canada [4, 5]. Similarly, future healthcare costs including drugs costs in the European Union have been estimated according to various scenarios based on demography, health status, and income [6]. Despite the plethora of research, however, no evidence exists on how to predict pharmacy service fees at the population level.

The changing healthcare environment in Korea makes it difficult to forecast the annual budget for pharmacy service fees and therefore determine dispensing fees for the following year through negotiations between the payer and pharmacist association [7, 8]. From the payer’s perspective, a more predictable reimbursement system for pharmacy service fees should be established to stabilize the health insurance budget [9]. From the pharmacist’s perspective, an adequate payment mechanism for dispensing services is needed based on a prediction model for pharmacy service fees [10].

Models that forecast healthcare costs commonly rely on a combination of factors including demographics (typically age and sex), diagnoses, and prescription drug utilization data [11]. In particular, developing a model to project pharmacy service fees should be preceded by an analysis of pharmacy services by pharmacy characteristics to consider the various factors influencing such fees. No prediction model of pharmacy service fees has thus far been developed in Korea. Based on the foregoing, this study compares the status of pharmacy services and projects annual pharmacy service fees by evaluating a model for forecasting based on pharmacies’ insurance claims data.

## Methods

### Database

This study used the national health insurance database of all insurance enrolees and medical aid beneficiaries in Korea [12]. We extracted a 1% sample of all pharmacies’ insurance claims data in 2012 through stratified systematic sampling and classified the data based on a pharmacy and a prescription for a descriptive analysis on dispensing status. We constructed the dataset to project pharmacy service fees by extracting dispensing data of all pharmacies every year from 2006 to 2012 from the nationwide health insurance claims database. The dataset was composed of time-series cross-section data that gathered according to the flow of time by quarter.

### Classification of pharmacies

To analyse differences in pharmacy service fees, pharmacies were classified based on the type of medical institution that issues over 80% of the pharmacy’s prescriptions: general hospital outpatient pharmacy [13], special hospital outpatient pharmacy [13], clinic outpatient pharmacy, and others. For example, if general hospitals issued over 80% of all prescriptions dispensed by a pharmacy, the pharmacy was classified as a general hospital outpatient pharmacy. A pharmacy was categorized as ‘others’ if no single type of medical institution makes up more than 80% of its prescriptions. Based on the grouping, three dummy variables were constructed for the regression analysis.

### Construction model to project pharmacy service fees

We constructed a model to project pharmacy service fees which comprises the following variables through a factor analysis associated with changes in annual pharmacy service fees: the number of prescriptions per pharmacy, the average dispensing days of prescriptions per pharmacy, the proportion of patients 65 years and older, a conversion factor, change of policy, and pharmacy type. The conversion factor is an index that applies every year to the dispensing fee agreement between the government and pharmacist association [14]. Change of policy represents whether the government changed the dispensing fee control policy or not; a dummy variable was constructed (no change = 0, change = 1).

### Four scenarios to project fees in 2013

To project pharmacy service fees in 2013, we assumed four scenarios. Scenarios 1 and 2 applied the average increase rate of annual pharmacy service fees in the past 7 years (2006–2012) and 3 years (2010–2012), respectively. Scenarios 3 and 4 applied the maximum and minimum values, respectively, for the rate of increase in the number of prescriptions in 2006–2012.

### Statistical analysis

By using the 2012 data, we performed a descriptive analysis to describe the differences in pharmacy services across pharmacy types. We analysed the average number of pharmacists, number of prescriptions, annual pharmacy service fees per pharmacy, and average drugs costs per prescription by pharmacy type for examining cost changes in pharmacy service fees.

By using the time-series cross-section data, we conducted a regression analysis to test the determinants of annual pharmacy service fees per pharmacy per quarter and to analyse changes in annual pharmacy service fees. The independent variables included the number of prescriptions per pharmacy, average dispensing days of prescriptions per pharmacy, proportion of patients aged 65 years and older, conversion factor, change of policy, and pharmacy type. The amounts of pharmacy service fees forecasted for 2013 were compared among the aforementioned four scenarios.

All statistical analyses were performed by using the SAS statistical application program (Version 9.4, SAS Institute Inc., Cary, NC, USA). The approval of the Institutional Review Board for this study with the general population was waived under Article 16 of the Rule of the Bioethics and Safety Act in Korea [15].

## Results

### Dispensing status by pharmacy type

Of the 21,283 pharmacies identified, 5.0% (1064), 4.6% (974), and 77.5% (16,340) were general hospital, special hospital, and clinic outpatient pharmacies, respectively, in 2012. The average number of pharmacists per pharmacy was 1.36, with the largest number (2.39) being in general hospital outpatient pharmacies. Clinic outpatient pharmacies dispensed the largest number of prescriptions (*n* = 3,869,364, 80.2%). General hospital outpatient pharmacies had a higher average daily number of prescriptions (111.9), annual dispensing fees ($25,546,342), and annual drugs costs ($215,728,000) than any other pharmacy. Pharmacy service fees ($7.2), average dispensing days (35.2), and average drugs costs ($60.6) were higher for general hospital outpatient pharmacies than for any other pharmacy (Table 1).

**Table 1:** Characteristics of pharmacies by type of medical institution in 2012

Category		All pharmacies	General hospital outpatient pharmacy^c^	Special hospital outpatient pharmacy^d^	Clinic outpatient pharmacy^e^	Others^f^
Number of pharmacies, n (%)		21,283 (100.0)	1064 (5.0)	974 (4.6%)	16,502 (77.5)	2743 (12.9)
Average number of pharmacists ± SD^b^		1.36 ± 0.78	2.39 ± 1.67	1.46 ± 0.87	1.32 ± 0.66	1.21 ± 0.57
Number of prescriptions, n (%)		4,824,860 (100.0)	378,512 (7.8)	256,292 (5.3)	3,869,364 (80.2)	320,692 (6.6)
Per pharmacy	Daily average number of prescriptions 100 ± SD ^b^	75.6 ± 70.9	118.6 ± 99.2	87.7 ± 93.1	78.6 ± 67.1	32.0 ± 54.3
	Pharmacy service fees per year 100 ± SD ^a b^	115,936.7 ± 113,175.9	255,463.4 ± 226,645.2	137,114.8 ± 135,937.0	114,829.4 ± 95,924.6	63,655.3 ± 85,875.8
	Drugs costs per year 100 ± SD ^a b^	343,518.2 ± 761,571.8	2,157,280.0 ± 2,588,749.4	396,526.1 ± 400,011.9	244,839.5 ± 246,262.2	214,662.6 ± 360,265.4
Per prescription	Pharmacy service fees ± SD ^a b^	5.1 ± 8.8	7.2 ± 9.1	5.2 ± 8.9	4.9 ± 6.5	5.4 ± 10.1
	Average number of prescription drugs ± SD ^b^	3.77 ± 3.58	3.48 ± 3.59	3.77 ± 3.76	3.82 ± 3.55	3.55 ± 3.91
	Average dispensing days ± SD ^b^	11.4 ± 12.3	35.2 ± 30.7	10.9 ± 12.4	8.8 ± 10.2	14.3 ± 18.5
	Average drugs costs ± SD ^a b^	15.2 ± 16.6	60.6 ± 70.1	15.1 ± 16.7	10.4 ± 12.4	18.4 ± 20.1

^a^Costs in Korean won were converted into US$ by using the conversion rate of 1200 won/US$

^b^Standard deviation

^c^Pharmacy that obtained more than 80% of prescriptions from general hospitals

^d^Pharmacy that obtained more than 80% of prescriptions from special hospitals

^e^Pharmacy that obtained more than 80% of prescriptions from clinics

^f^Pharmacy that was not c, d, or e

### Regression model to project pharmacy service fees per pharmacy per quarter

The time-series cross-section data analysis estimated that the quarterly pharmacy service fees per pharmacy increased by $4.7 for one additional prescription per pharmacy. Pharmacy service fees increased by $307.3 for one additional dispensing day per prescription and by $1082.3 for a 1% increase in the proportion of patients aged 65 years and older in the same period. Pharmacy service fees increased by $494.8 for one additional point of the conversion factor and decreased by $956.1 for a change of policy. Compared with clinic outpatient pharmacies, the fees of general hospital and special hospital outpatient pharmacies increased by $6028 and $1140.0, respectively, while those of other pharmacies decreased by $184.1 (Table 2).

**Table 2:** Factor analysis for projection using time-series cross-section data on pharmacy service fees per pharmacy per quarter

Category	Coefficient^a^	SD^b^	t	95% CI		*p*-value
Number of prescriptions per pharmacy per quarter	4.7	0.002	2.229	4.667	4.674	< 0.001
Average dispensing days of prescriptions per pharmacy per quarter	307.3	1.467	0.175	304.4	310.2	< 0.001
Ratio of patients aged 65 years and older	1082.3	70.193	0.013	944.8	1219.9	< 0.001
Conversion factor	494.8	2.056	0.201	490.7	498.8	< 0.001
Change of policy	−956.1	11.767	−0.068	− 979.2	− 933.1	< 0.001
Hospital type						
General hospital outpatient pharmacy ^c^	6028.2	56.402	0.089	5917.6	6138.7	< 0.001
Special hospital outpatient pharmacy ^d^	1140.0	39.156	0.024	1063.2	1216.7	< 0.001
Clinic outpatient pharmacy ^e^	− 1494.5	101.889	−0.012	− 1694.2	− 1294.8	< 0.001
Other ^f^	−184.1	18.888	−0.008	− 221.2	− 147.1	< 0.001
Constant	−34,916.7	125.224	−0.232	−35,166.7	− 34,666.7	< 0.001

^a^Costs in Korean won were converted into US$ by using the conversion rate of 1200 won/US$

^b^Standard deviation

^c^Pharmacy that obtained more than 80% of prescriptions from general hospitals

^d^Pharmacy that obtained more than 80% of prescriptions from special hospitals

^e^Pharmacy that obtained more than 80% of prescriptions from clinics

^f^Pharmacy that was not c, d, or e

### Projection of pharmacy service fees in 2013 according to the four scenarios

**Fig. 1:**
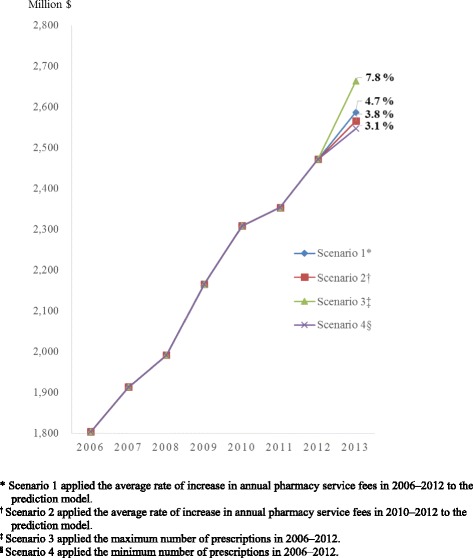
Pharmacy service fees in 2013 estimated by the prediction model

The pharmacy service fees of all pharmacies in 2013 were forecasted to be $2587.4 million (Scenario 1), $2565.8 million (Scenario 2), $2663.5 million (Scenario 3), and $2547.0 million (Scenario 4) (Table 3). Compared with 2012, the rate of increase in the pharmacy service fees of all pharmacies in 2013 was estimated to be between 3.1% (Scenario 4) and 7.8% (Scenario 3) (Fig. 1).

**Table 3:** Pharmacy service fees of the next year (2013), using the regression model for projection

Category	Pharmacy service fees for a quarter ^a, c^	Pharmacy service fees in 2013^a,b, d^	Pharmacy service fees in 2012^a,b, d^	Year-on-year increase rate ^e^,%
Scenario 1 ^f^	31,744.4	2587.4	2470.8	4.7
Scenario 2 ^g^	31,643.8	2565.8	2470.8	3.8
Scenario 3 ^h^	32,678.1	2663.5	2470.8	7.8
Scenario 4 ^i^	31,248.2	2547.0	2470.8	3.1

^a^ Costs in Korean won were converted into US$ by using the conversion rate of 1200 won/US$

^b^ Million US$

^c^ Pharmacy service fees per pharmacy

^d^ Pharmacy service fees of all pharmacies

^e^ Increase rate of the prediction value of 2013 on 2012

^f^ Applied the average increase rate of annual pharmacy service fees in 2006–2012

^g^ Applied the average increase rate of annual pharmacy service fees in 2010–2012

^h^ Applied the maximum number of prescriptions in 2006–2012

^i^ Applied the minimum number of prescriptions in 2010–2012

## Discussion

We developed a model to project pharmacy service fees per pharmacy per quarter by identifying the factors associated with these fees and by including forecasted values for the variables. Time-series cross-section data were analysed to determine the factors that influence pharmacy service fees, using the health insurance claims data submitted by all pharmacies between 2006 and 2012.

The ratio of pharmacy was the highest for clinical hospital outpatient pharmacies among all pharmacies in 2012. While general hospital outpatient pharmacies that had higher pharmacy service fees, average dispensing days, and average drugs costs per prescription had higher annual pharmacy service fees ($25,546,342) and annual drugs costs ($215,728,000) per pharmacy than any other pharmacy.

The rate of increase in the pharmacy service fees of all pharmacies between 2012 and 2013 was projected to be between 3.1% (Scenario 4) and 7.8% (Scenario 3). This finding is consistent with previous reports in Korea [16] and Australia [17] but not with those in the United Kingdom [18]. The average increase rate of annual pharmacy service fees was 5.4% within this range (3.1–7.8%) between 2005 and 2011 in Korea [16]. The increase rate of prescription volumes covered by the Pharmaceutical Benefits Scheme and Repatriation Pharmaceutical Benefits Scheme of Australia was projected to be 3.4–3.9% between 2016 and 2020 in sixth Community Pharmacy Agreement between the Commonwealth of Australia and the Pharmacy Guild of Australia despite methodological discrepancies [17, 19]. By contrast, the United Kingdom’s Community Pharmacy Contractual Framework funding package for 2016–2017 was reduced by about 4% compared with 2015–2016 [18]. In the United Kingdom, dispensing fee remuneration for prescriptions comprised the dispensing fees (essential and advanced services) and retained margins determined by using the margins survey [20]. The change of dispensing fee remuneration was due to a reduction in the retained margins by discounting drugs prices because dispensing fees (essential and advanced services) have remained steady every year [21]. On the contrary, no research has thus far predicted dispensing fees in the United States, which does not have a universal public healthcare system [19]. Only the results of a professional dispensing fee analysis for Medicare and Medicaid by state have been revealed [22].

Pharmacy service fees are a function of the price factor (i.e. conversion index) and the quantity factor (i.e. number of prescriptions dispensed) [23]. The conversion index established during fee negotiations between the payer and service providers (i.e. the Korean Pharmacist Association) every May is applicable to the model to project. As the number of prescriptions dispensed is dependent on the number of prescriptions and number of dispensing days per prescription, it is necessary to predict the number of prescriptions dispensed more precisely than the price factor. Pharmacy service fees are associated with a number of factors such as socioeconomic status, individual pharmacy characteristics, and time effects. As the time series data model takes into account individual pharmacy characteristics and time effects, it is regarded as suitable for analysing the factors influencing pharmacy service fees.

As the application of the model for forecasting may yield different results depending on the monitoring period and socioeconomic changes that influence demand for medical services, a mechanism to adjust for these factors is needed. For example, the length of a monitoring period needs to be established by consensus between the payer and service providers. In addition, it is necessary to accurately measure the variables for the longer-term management of dispensing fees. To accurately predict the number of prescriptions dispensed that have the greatest impact on dispensing fees, a mechanism to predict those factors influencing demand for medical services (e.g. population aging, economic development) should be developed in advance.

There was a huge difference in pharmacy service compensation systems between Korea and Australia. Pharmacists are compensated for dispensing services only without pharmacy margins for purchased drugs in Korea, whereas pharmacy service fees include dispensing fees and the pharmacy mark-up in Australia. In Australia, dispensing fee remuneration for prescriptions is composed of dispensing fees and administration, handling, and infrastructure fees (replaces the pharmacy margin) [24]. The latter were introduced to replace the previous pharmacy mark-up and delink remuneration from the price of a medicine to allow changes to pricing policy that will not have a significant impact on pharmacy remuneration in the sixth CPA on 1 July 2015 [25].

In Australia, pharmacy service fees were set by consensus between the government and pharmacist association every 5 years based on the future five-year prediction [26]. The management range was 95.0–105.0% of the predicted dispensing between 2006 and 2010 [27]. In the United Kingdom, pharmacy service fees are also determined by annual negotiations between the government and pharmacist association; the compensation for annual pharmacy services includes drug margins [28]. By adopting the Australian model, the payer (i.e. the National Health Insurance Service) and service providers (i.e. the Korean Pharmacist Association) would be able to predict pharmacy service fees in the following year by mutual consensus, and set a management range at the level that enables both parties to share the risk. If the forecasted amount deviates from the actual amount beyond the management range, considerations could be given during fee negotiations in the subsequent year, such as the adjustment of the conversion index.

We showed that general hospital outpatient pharmacies received higher pharmacy service fees than clinic outpatient pharmacies, suggesting that these hospitals issued a greater number of prescriptions. This finding is consistent with that of a previous study [29]. The number of prescriptions dispensed is dependent on physicians’ prescribing activity. Therefore, a methodology that analyses the treatment behaviour of the departments that issue prescriptions should be developed to project annual dispensing fees more accurately.

Whereas previous studies developed a projection model of pharmacy service fees based on the rate of change for each variable by using claims data such as diagnoses and medications and validated projection models of pharmacy service fee by comparing R^2^ values of the models [3, 11, 30], the projection model developed in this study is effective for validating the differences in projected and observed pharmacy service fees. Pharmacy service fees in Korea include fees for service provided by the pharmacy and pharmacist but not drug margin. The pharmacy service fees vary according to the type of medical institution the patient uses. For example, a prescription issued by a bigger institution such as general hospital has a greater number of prescribed days, resulting in greater pharmacy service fees. Therefore, to examine cost changes as in the existing studies, we analyzed pharmacy service fees per prescription. To analyze changes in service fees, we projected future pharmacy service fees according to the type of pharmacy which was based on the source of service fees. In validation tests between 2009 and 2012 (results not shown), these differences were estimated to be − 2.5% to 3.8%. Drug management fees were reduced considerably in 2011 (− 2.5%) and the number of prescriptions dispensed decreased due to the economic recession [31]. These changes may have underestimated the projected values for 2010–2012 as well as the rate of increase in dispensing fees in 2013 compared with the scenarios that applied data from the preceding 7 years.

Our study has several limitations. First, the pharmacies included in our analysis may not be representative of all community pharmacies in Korea. The number of prescriptions dispensed at pharmacies near general hospitals was lower than the national average. Follow-up research using a nationally representative sample may thus be beneficial for the effective management of the health insurance budget. In addition, it was difficult to accurately classify pharmacies; pharmacy types may therefore have been misclassified.

## Conclusions

We showed that general hospital outpatient pharmacies received more annual pharmacy service fees than any other pharmacy because of their higher daily average number of prescriptions per pharmacy. Our findings suggest that future pharmacy service fees can be managed by using a regression model, which can serve as a useful tool when negotiating dispensing fees between the payer and pharmacist association. The change in the projection of annual pharmacy service fees in Korea is similar to that in Australia but not that in the United Kingdom.

## Abbreviations

CPA: Community pharmacy agreement
SD: Standard deviation
